# Prevalence of peripheral neuropathy and its associated demographic and health status characteristics, among people on antiretroviral therapy in Rwanda

**DOI:** 10.1186/1471-2458-14-1306

**Published:** 2014-12-19

**Authors:** David Kabagema Tumusiime, Francois Venter, Eustasius Musenge, Aimee Stewart

**Affiliations:** Research Centre, College of Medicine and Health Sciences, University of Rwanda, Box 3286, Kigali, Rwanda; Wits Reproductive Health and HIV Institute (Wits RHI), Department of Medicine, University of Witwatersrand, Johannesburg, South Africa; Division of Biostatistics and Epidemiology, School of Public Health, Faculty of Health Sciences, University of Witwatersrand, Johannesburg, South Africa; Department of Physiotherapy, School of Therapeutic Sciences, University of Witwatersrand, Johannesburg, South Africa

**Keywords:** Peripheral neuropathy, Antiretroviral therapy, Rwanda

## Abstract

**Background:**

The introduction of antiretroviral therapy (ART) has dramatically reduced the mortality rate of people living with HIV (PLHIV). However, complications of both HIV and ART, such as peripheral neuropathy currently affect PLHIV. The purpose of this study was to establish the prevalence of peripheral neuropathy of the lower extremity and, its association with demographic and health status, characteristics among people on ART in Rwanda.

**Methods:**

A cross sectional study was conducted among 507 women and men aged between 18 and 60 years, on ART, randomly selected from eight selected ART clinics in Rwanda. Brief Peripheral Neuropathy Screen was used to assess peripheral neuropathy.

**Results:**

Peripheral neuropathy prevalence was 59% overall, mean age of the participants was 39.7 (±9.2) and a slightly older age was associated with peripheral neuropathy; [42(±9.2) vs 37 (±8.8) (p < 0.001)]. 78% of participants living in urban settings compared to 40% in rural settings reported peripheral neuropathy, 69% of participants with higher levels of education (secondary level and above) reported lower extremity neuropathy.

The three factors were significantly associated with peripheral neuropathy in multivariable model analysis: older age [aOR = 1.1, 95% CI (1.0, 1.2), p < 0.001], primary education level [aOR = 0.6 95% Cl (0.3, 1.0), p = 0.04] and urban setting [aOR = 0.1, 95% CI (0.06, 0.3), p < 0.001], after adjusting for other factors. None of the health status characteristics namely; the level of CD4 cell count, duration of HIV infection and duration on ART, was independently associated with peripheral neuropathy.

**Conclusions:**

The prevalence of peripheral neuropathy among PLHIV on ART in Rwanda is high. It is unclear why urban setting has an effect on PN levels in this cross sectional study, but does suggest that unidentified social and lifestyles factors may have a role in subjective symptoms and objective signs, of PN.

## Background

HIV/AIDS continues to cause high mortality and morbidity in many sub – Saharan African countries [1]. The introduction of antiretroviral combination therapy has dramatically reduced the mortality rate of people living with HIV [2, 3]. However, with substantially expanded life expectancy and the long-term use of antiretroviral therapy, complications such as peripheral neuropathy (PN) are the most prevalent neurological manifestations now seen in HIV/AIDS [4]. Peripheral neuropathy commonly affects people’s daily function and quality of life (QoL) among populations with HIV infection [5]. In addition, the PN is likely enhanced by various demographic and health status characteristics, among the PLHIV [6]. Existing data on prevalence of peripheral neuropathy is poor and there is no known data as to how peripheral neuropathy is associated with different demographic and health status characteristics of PLHIV in Rwanda, thus, limiting an early and appropriate health care intervention such as physiotherapy. The purpose of this study was to establish the prevalence of PN of the lower extremity, and the associated demographic and health status characteristics, among PLHIV on ART in Rwanda.

## Methods

### Study design, participants and setting

A cross sectional study was conducted among adult men and women participants aged 18 and above, with HIV infection and on ART. In Rwanda since 2011, all people with HIV and who have less than 350 m//m^3^ of CD4 cell counts, are put on ART. The first line regimen contains Tenofovir + Emtricitabine/Lamivudine + Nevirapine/Efavirenz or Abacavir + Lamivudine + Nevirapine/Efavirenz (TDF + FTC/3TC + NVP/EFV or ABC + 3TC + NVP/EFV). However, a few cases might still take d4T in the first line depending on the health status of the patient [7].

Clinical information from the patients’ medical files, about the pathologies which might be associated with peripheral neuropathy, was assessed. From the information, the participants with known active opportunistic infection such as TB or others, disorders of the central nervous system, history of diabetes, Vitamin B12 deficiency, renal failure, hypothyroidism and other pathologies, were excluded from the study sample. The participants were identified from eight randomly selected public ART clinics from all four provinces and the city of Kigali, in Rwanda. Participants attended the clinics for routine health care management that included; receiving ARV medication, health care consultations, counselling and other advice, and laboratory testing including CD4 cell count monitoring. A systematic random sampling was used to invite volunteers for participation. The pilot study [7] prior to this study indicated that this sampling procedure was feasible and practical. Therefore, a list of potential participants was made at every visit at each selected clinic and the process was repeated each day for a period of two weeks. The total number of potential participants (N) on the list made for each visit was multiplied by 20% to get the sample (n) of participants on each visit/day (n = N*20%). The study population was considered as a homogeneous population, thus the systematic random selection was done by randomly choosing the first person between 1 and K, then taking every K^th^ number thereafter, where K^th^ was a sampling interval on the ordered list made for a day. The K^th^ was calculated by dividing the total number of the participants on the list (N), by the sample size to be selected per day (n); (K^th^ = N/n), [8]. The procedure was repeated for a period of two weeks (10 working days) which was the maximum period designated for data collection at each selected ART clinic. A total number of 3180 PLHIV were registered to have attended the eight selected ART clinic services within the two week period of the study. Using the procedure described above, a sample of 636 potential participants was systematically and randomly invited from the total registered number (3180). The whole process of data collection at the eight selected ART clinics took 16 weeks (two weeks at each clinic) from March to July 2012.

An ethical clearance certificate (protocol number M080812) was obtained from the Human Research Ethics Committee at the University of the Witwatersrand. As the data were collected in Rwanda, national ethical clearance (No. 032IRB052011) was also obtained from the Institutional Review Board at Kigali Health Institute and the proposal was scientifically approved by the National Commission for Control of HIV and AIDS (approval letter No. 0137/CNLS/2011/S.E), in Rwanda. An information letter explaining the details of the study and inviting participants to participate in it was given to them prior to their recruitment into the study. Participants signed a consent form agreeing to their participation and giving their permission to use their medical records/files.

### Recording of demographic and health status, characteristics

The demographics captured included age, gender, level of education, marital status, and place of residence, namely; a rural or urban setting. Health status information included the duration of HIV infection, current ARV regimen combination, duration on ART, other pathologies (the ones listed in the exclusion criteria above) that could cause PN, the most recent CD4 cell count level, and whether the participant presented PN symptoms (PNS) prior to starting on ART or afterwards. We gathered the information from the participants themselves and from their medical file records. The medical file records considered were on papers (hard copies) and those with missing needed data were excluded. About 21 file records had some missing data and these were replaced with additional file of the participants (after screening them for inclusion criteria) with complete records to avoid reduced sample size for the power calculation. The checklist was completed by trained staff together with the participant, and assistance was sought when needed from the medical practitioner working at the respective outpatient ART clinic.

### Assessment of peripheral neuropathy

A brief peripheral neuropathy screen (BPNS), which is a validated tool to assess HIV – associated peripheral neuropathy [9] was used to assess the PN in Rwanda in a prior study [10], and thus was again used to assess PN in this study. The BPNS assesses subjective symptoms and objective signs, of PN. Three assessors; DT, JT and JM who are senior physiotherapists did the assessments using the guidelines and principles described in other studies [5]. All the assessors were blinded to any clinical symptoms and signs of the participant, before start on ART. The assessment included; asking participants to rate the presence and severity of symptoms for each leg separately, using a scale of 1 (mild) to 10 (severe). Symptoms included pain, aching, or burning in feet and/or legs, pins and needles in feet and/or legs and numbness in feet and/or legs. The single highest of the six scores (three for each leg) was then converted to a subjective peripheral neuropathy grade as follows: symptoms absent = grade 0, score 1–3 = grade 1, score of 4–6 = grade 2, and score of 7–10 = grade 3. Symptoms did not have to be bilateral to be graded as ≥ 1. Objective findings included in the BPNS were loss of vibration perception and abnormal ankle deep tendon reflexes. Vibration perception was evaluated using a 128-Hz tuning fork, maximally struck and applied to the distal interphalangeal joint of the hallux of each foot. Vibration sense was defined as normal for a vibration felt for > 10 seconds, mild loss for 6–10 seconds, moderate loss for ≥ 5 seconds, and severe loss for no feeling of vibration. Ankle reflexes were defined as absent, hypoactive, normal, hyperactive, or clonus. The primary outcome of interest in this measure was the presence of PN, which was defined as the combination of at least one subjective neuropathy grade greater than 0 and either abnormal vibratory sense or ankle deep tendon reflex, bilaterally, as has been defined and used in previous studies [5, 9].

### Statistical analysis

Descriptive analysis; tabulations for frequencies and percentages for categorical variables and measures of central tendency (means) and measures of variability (standard deviations) for continuous variables, were used. A bivariate analysis using Pearson’s Chi – square or appropriate Fisher’s exact tests was used when analysing the prevalence distribution of PN and its association with categorical demographic and health status variables. The demographic variables included gender, level of education, occupation, marital status and place of residence. Health status variables included the duration of HIV infection, current ARV regimen combination, duration on ART, the most current CD4 count and presence of PN symptoms and onset date of PN symptoms. The student’s t-test (or Mann Whitney for non-normally distributed data) was used for pairs of continuous variables.

Variables which were statistically significant in a univariate model were further included into the multivariate model with logistic regression analysis, to establish the association of PN and its magnitude (odds ratio), upon controlling of confounding factors. The analysis was done using STATA (version 11, Stata Corp, College Station, Texas, USA) and the level of significance was set at 5%, hence statistical significance was achieved when p < 0.05.

## Results

### Participants and prevalence of peripheral neuropathy

With the sample of six hundred and thirty six (636) potential participants randomly invited to participate in the study, five hundred and seven (507), equivalent to 80% of the sample were eligible according to the set inclusion criteria mentioned in the method section. The participants completed the outcome measures and their data were eligible for analysis. Out of the analysed sample of 507, three hundred (300/507) equivalent to 59% [95% CI (54%, 63%)] were diagnosed with PN using the BPNS.

### Peripheral neuropathy and demographic characteristics

The distribution and association of PN with the demographic characteristics is shown in Table 1 with the mean age of the participants being 39.7 (SD ±9.2). Participants with PN were slightly older than those without PN [42(SD ± 9.2) vs 37 (±8.8) (p < 0.001)]. PN was more predominant (69%) among participants with a higher level of education (p < 0.01). With the chi-square for categorical variables, there was a statistically significance (p < 0.001) difference in distribution of PN among the participants across the occupational activities. The difference in distribution of PN with marital status was significant (p = 0.003) also. Peripheral neuropathy among participants living in the urban setting was 78% compared to 40% among the ones living in the rural setting (p < 0.01).

**Table 1:** **Distribution of PN and its association with demographic characteristics**

		PN n (%) n = 507			
Variable		No PN (n = 207)	PN (n = 300)	p-value	All
**Age (mean ± SD)**		37 (±8.8)	42 (±9.2)	*<0.001*	39.8 (±9.2)
**Gender**	Female	156 (43)	210 (57)	*0.402*	366 (73)
	Male	51 (37)	87 (63)		138 (27)
**Education**	None	39 (35)	73 (65)	0.002*	112 (22)
	Primary	123 (49)	128 (51)		251 (50)
	> = Secondary	45 (31)	99 (69)		144 (28)
**Occupation**	Public employed	13 (31)	29 (69)	<0.001*	42 (8)
	Peasant/Farmer	118 (53)	106 (47)		224 (44)
	Self employed	33 (37)	56 (63)		89 (18)
	Unemployed	43 (28)	109 (72)		152 (30)
**Marital status**	Single	17 (49)	18 (51)	0.003*	35 (7)
	Married	119 (52)	112 (48)		231 (46)
	Divorced/Separated	18 (22)	63 (78)		81 (16)
	Window/Widower	53 (33)	107 (67)		160 (31)
**Setting/Residence**	Urban	58 (22)	200 (78)	<0.001*	258 (51)
	Rural	149 (60)	99 (40)		248 (49)

*Denotes significant association, SD = Standard deviation, n = number of participants.

### Peripheral neuropathy and health status characteristics

Table 2 shows that there was no significant difference (p = 0.98) in distribution of PN among participants with different CD4 cell count levels. A statistically significant difference (p = 0.002) was found in the distribution of PN among participants with different durations of HIV infection. PN appeared in 73% of participants who had the infection for the last seven and above years. Similarly, a longer duration on ARVs was associated with PN (p = 0.001), which was predominantly observed in the period of 4 – 6 years, but with almost 10% less among those of 7 years and above. Table 3 reveals that almost all PN characteristics were significantly associated (p < 0.001) with the participants’ settings, where the PN appeared more severe among the urban dwellers.

**Table 2:** **Distribution of PN and its association with health status characteristics of the participants**

		PN n (%) n = 507			
Variable		No PN	PN	p-value	All
**CD4 cell count**	<=350	63 (51)	91 (49)	*0.98*	154 (30)
	>350	144 (41)	209 (59)		353 (70)
**Duration since HIV diagnosis**	0 – 3 years ago	72 (46)	84 (54)	<0.001*	156 (31)
	4 – 6 years ago	91 (49)	96 (51)		187 (37)
	7 and above yrs	44 (27)	120 (73)		164 (32)
**Current ARV regimen**	Non d4T containing	156 (39)	247 (61)	0.056	403 (79)
	d4T containing	51 (49)	53 (51)		104 (21)
**Duration on ARVs (in years)**	0 – 1	71 (58)	52 (42)	<0.001*	123 (24)
	1 – 3	58 (36)	101 (64)		159 (32)
	4 – 6	61 (30)	108 (64)		169 (33)
	7 and above	17 (41)	39 (70)		56 (11)
**ARV regimen changes**	None	44 (43)	48 (57)	*0.045*	102 (20)
	Once	120 (44)	150 (56)		270 (53)
	Two and more	43 (32)	92 (68)		135 (27)
**ARV started with**	Non D4T containing	62 (37)	104 (63)	0.27	166 (33)
	D4T containing	145 (43)	196 (57)		341 (67)

*Denotes significant association, n = number of participants.

**Table 3:** **Prevalence of PN characteristics among the participants in urban and rural settings**

PN characteristic measures		Settings n (%) n = 507		
		Urban	Rural	p-value
**PN in the feet**	Absent	81 (33)	129 (52)	<0.001*
	Present	167 (67)	119 (48)	
**PNS severity**	None	31 (13)	130 (52)	<0.001*
	Mild	48 (20)	27 (11)	
	Moderate	78 (32)	40 (16)	
	Severe	88 (36)	47 (19)	
**PNS distribution**	None	34 (13)	134 (54)	<0.001*
	Feet only	74 (29)	46 (20)	
	Ankle to knee	95 (37)	66 (29)	
	Above knee	55 (21)	48 (21)	
**Sense of vibration**	Reduced/absent	213 (83)	98 (40)	<0.001*
	Normal	45 (17)	150 (60)	
**Ankle reflex**	Reduced/absent	190 (74)	94 (38)	<0.001*
	Normal	68 (26)	154 (62)	

*Denotes significant p-value for the association.

### The demographic and health status characteristics predicting the prevalence of Peripheral Neuropathy

Univariate analysis in Table 4, demonstrates that PN is associated with demographic characteristics such as age [OR = 1.1, 95% CI (0.1, 1.2) p < 0.001], level of education [OR = 0.6, 95% Cl (0.4, 0.9) p < 0.01], occupation (being peasant or a farmer compared to public employed) [OR = 0.4, 95% CI (0.2, 0.8) (p < 0.01], marital status [OR = 3.3, 95% CI (1.0, 4.0) p < 0.01] and with rural versus urban setting [OR = 0.2, 95% CI (0.1, 0.3) p < 0.001]. Health status characteristics predicting PN include duration of HIV infection [OR = 2.3, 95% CI (1.5, 3.7) p = 0.001], and the duration on ART [OR = 2.4; 95% CI (1.5, 3.8) (p < 0.001)], 95% CI (1.5, 3.9); (p < 0.001)] and ≥ 7 years [OR = 3.1; 95%CI (1.6, 6.1). All significantly associated characteristics with PN in the univariate analysis were put into the multivariate model for further analysis. The model revealed that some characteristics were independently associated with the PN. These include age [aOR = 1.06, 95% CI (1.03, 1.08) p < 0.001], level of education [aOR = 0.6, 95% Cl (0.3, 1.0) p = 0.04], some categories of the duration on ART [1-3 years = aOR: 2.6, Cl (1.4, 4.8) p < 0.001, 4-7 years = aOR: 2, Cl (1.0, 4.1) p < 0.05] and urban versus rural setting [aOR = 0.1, 95% CI (0.06, 0.3) p < 0.001]. Thus, age, level of education, duration on ART and the urban versus rural setting were the major predictors of PN in this study.

**Table 4:** **Univariate and multivariate analysis for the associated and predicting demographic and health status characteristics, to PN**

Characteristics		Prevalence (%) of PN (n = 300)	Univariate models		Multivariate models	
			Odds ratio (OR) (95% CI)	p-value	Adjusted odds ratio (aOR) (95% CI)	***p-value***
**Age**		42 (±9.2)	1.1 (1.0, 1.1)	<0.001	1.1 (1.0, 1.1)	<0.001*
**Gender**	Female	210 (57)	1.0 (ref)	0.25		
	Male	87 (63)	1.3 (0.8, 1.9)			
**Education**	None	73 (65)	1.0 (ref)		1.0 (ref)	
	Primary	128 (51)	0.6 (0.4, 0.9)	0.01	0.6 (0.3, 1.0)	0.04*
	> = Secondary	99 (69)	1.2 (0.7, 2.0)	0.55	0.6 (0.3, 1.3)	0.2
**Occupation**	Public employed	29 (69)	1.0 (ref)		1.0 (ref)	
	Peasant/Farmer	106 (47)	0.4 (0.2, 0.8)	0.01	0.9 (0.3, 2.2)	0.76
	Self employed	56 (63)	0.8 (0.3, 1.7)	0.5	0.3 (0.1, 0.9)	0.03
	Unemployed	109 (72)	1.1 (0.5, 2.4)	0.74	0.6 (0.2, 1.4)	0.22
**Marital status**	Single	18 (51)	1.0 (ref)			
	Married	112 (48)	0.9 (0.4, 1.8)	0.75		
	Divorced/Separated	63 (78)	3.3 (1., 4)	<0.01		
	Window/Widower	107 (66)	1. 9 (0.9, 4.)	0.09		
**Setting/Residence**	Urban	200 (78)	1.0 (ref)		1.0 (ref)	
	Rural	99 (40)	0.2 (0.1, 0.3)	<0.001	0.1 (0.06, 0.3)	<0.001*
**CD4 cell count**	<=350	91 (49)	1.0 (ref)			
	>350	209 (59)	1.0 (0.7, 1.5)	1.008		
**Duration since HIV diagnosis**	0 – 3 years ago	84 (54)	1.0 (ref)		1.0 (ref)	
	4 – 6 years ago	96 (51)	0.9 (0.6, 1.4)	0.60	0.6 (0.3,1.1)	0.08
	7 and above yrs	120 (73)	2.3 (1.5, 3.7)	<0.001	1.4 (0.7, 3.0)	0.33
**Current ARV regimen**	Non D4T containing	247 (61)	1.0 (ref)			
	D4T containing	53 (51)	1.0 (0.4, 1.0)	0.06		
**Duration on ARVs (in years)**	0 – 1	52 (42)	1.0 (ref)		1.0 (ref)	
	1 – 3	101 (64)	2.4 (1.5, 3.8)	<0.001	2.6 (1.4, 4.8)	0.001
	4 – 6	108 (64)	2.4 (1.5, 3.9)	<0.001	2.0 (10, 4.1)	0.05
	7 and above	39 (70)	3.1 (1.6, 6.1)	0.001	2.2 (0.8, 5.9)	0.1
**ARV regimen changes**	None	48 (57)	1.0 (ref)			
	Once	150 (56)	0.9 (0.6, 1.5)	0.80		
	Two and more	92 (68)	1.6 (1.0, 2.8)	0.08		
**ARV started with**	Non D4T containing	104 (63)	1.0 (ref)			
	D4T containing	196 (57)	0.8 (0.6, 1.2)	0.27		

*Denotes significant p-value for the association of the characteristics with PN in a multivariate model, CI = Confidence Interval.

## Discussion

HIV has changed from being a terminal illness to a chronic condition in the ART era [11] and PLHIV are now living longer. However, living with HIV and on ART has complications that include PN which are disabling [12]. Peripheral neuropathy has been reported as one of the common neurological complications of HIV infection in the ART era [4, 13]. HIV – associated neuropathy is reported as a cause of morbidity among PLHIV due to the effects on their quality of life [13, 14]. This study identified the prevalence of PN and its association with demographic and health status, characteristics of PLHIV on ART from selected rural and urban ART clinics in Rwanda.

The overall prevalence of PN was very high (59%) in this study compared to previous studies conducted in Rwanda. However, the prevalence of 40% demonstrated in this study in rural dwellers is the same as the previous study in Rwanda [15] that found a prevalence of 40.5% from one rural district hospital. But, the previous study was conducted in only one rural district hospital and had a smaller sample size, compared to our study that covered four selected rural health facilities. Overall, our study was conducted in eight health facilities selected from both rural and urban settings and with increased sample size. Secondly, our study used a more rigorous assessment instrument for the PN; the BPNS. The previous study [15] used the Subjective Peripheral Neuropathy Screen (SPNS) that evaluates peripheral neuropathy symptoms (PNS) subjectively [16], unlike the BPNS that evaluates signs objectively in addition to the subjective assessment. The objective evaluation such as the deep tendon reflex and sense of vibration may detect neurological signs in asymptomatic participants [5]. The SPNS is unlikely to detect all patients with PN and will underestimate the prevalence of PN, as Evans et al., [6] reported that PN may frequently occur without symptoms, and PN may vary depending on the defined criteria used [12].

The prevalence in our study is higher than in most other studies in Africa, such as by Luma et al., [14] in Cameroon with 28%, and Mehta et al., [5] in Kenya with 36%. The prevalence of PN in our study is slightly lower than one in Brazil by Zanetti et al., [17] where the prevalence was 69.4%. Some factors that may explain the differences between our study and the one in Brazil [17], include the population’s average age, types of medications (ART regimen), diseases (HIV) stage, procedures to control PN, unidentified environmental factors and sample size, could all contribute to the findings of differing prevalence rates.

An older age was associated with the PN. This is similar to other studies in which age has been identified as a risk factor for developing PN [5, 6, 12, 18, 19]. Monitoring PN occurrence is important for possible and appropriate management in this group. The study by Haanpää and colleagues, [20] highlighted that early identification and management of the neuropathic pain symptoms is important in primary care to improve the quality of life.

This study indicated a higher prevalence of PN in people on ART living in urban than in rural settings in Rwanda. The very big difference between rural and urban rates is puzzling. A possible reason for the difference may be that people living in a Rwandan urban setting are more likely to be physically inactive [21]. Physical inactivity has been reported as a risk factor for neuromuscular conditions (which may include PN) in PLHIV [22]. Similarly, increased body weight is usually common among people who are physically inactive. This has been identified in one of the studies conducted in Rwanda as a risk factor for neuropathy, although we did not measure it [23]. Again, the literature shows that physical exercises reduces weight and other co-morbidities [24] such as lipodystrophy which is a potential mediator for PN in HIV [25]. Although this study did not assess weight and lipodystrophy, Mutimura et al., [26] indicated that lipodystrophy was more prevalent among PLHIV in urban than rural, settings in Rwanda. Routine monitoring and assessment of PN, physical inactivity, and body weight particularly in urban settings, would be useful to develop appropriate strategies for the management of PN among PLHIV.

The CD4 cell count was not associated with PN, contrary to what has been found in other studies done in Africa, such as the study by [27] in Burkina Faso, and the study by Luma et al. [14] in Cameroon. Our study was cross sectional and all participants were outpatients and most of them (70%) had CD4 count of 350 m//m^3^ and above. This might explain the differences as decreased CD4 cell count has been shown to be associated with co-morbidities including PN in PLHIV [28]. Similar findings of non associated decreased CD4 cell count with PN have been demonstrated in the study by Morgello et al., [29] and a study by Schifitto with colleagues [30]. This implies CD4 might not be a measure of risks once on ART [11].

Likewise, the d4T containing regimen was not associated with PN, which is contrary to the findings in other studies [27, 31, 32], however, is similar with the study by Luma et al. [14] which showed no association of d4T use with PN. A possible reason being that people who experience PN as result of d4T have it in the initial stages of the therapy [33, 34]. In their study, Hung and colleagues [33] demonstrated that the continuation of the use of the so called “d-drugs” such as d4T among patients who persist with PN from the initial stages did not show a significant difference between the use of non d-drugs, in worsening the neuropathy. It is common practice that in such cases of PN suspected to be as a result of d4T, the medication is usually discontinued. The experience of PN at later stages of therapy is likely attributable to other factors than d4T. On the other hand, because of the evidence that d4T has been associated with PN, the medication was reduced and eliminated in the ARV regimen in some countries (7) including Rwanda. Thus, few participants were on d4T containing regimen, of which less number might have statistical power to demonstrate the significant results.

The duration of HIV infection and duration on ART, did not independently predict the occurrence of PN after adjusting for other confounders in a multivariable model, with exception of the duration of 1 – 3 years that appeared a predictor of PN. To the authors’ knowledge, it is not yet clear to why being on ART in the first 3 years predicts PN than longer periods of 4 – 6 or 7 and above years. Further investigations into this with longitudinal study designs would be helpful in clarifying this. Otherwise, similar findings in other studies in Africa such as by Oshinaike et al. [12] indicated the trend of ART duration and non - association with PN. Some other studies however for instance the one by Robinson-Papp et al [35], highlighted the duration of HIV infection as a risk factor for PN. It is important to note that there is likelihood that the duration the patient has been HIV positive and on ART, may influence the occurrence of PN, but our study shows that the occurrence is influenced by the presence of other risk factors, such as duration on ART particularly during the period of 1 – 3 years on ART. It is likely the ART associated PN usually manifests during the first years on the ART [29], and reduces in later years, probably due to other health care interventions. However, this could not be confirmed with our cross sectional study, further studies with longitudinal design are warranted.

The strength of this study is demonstrated in the large sample size and inclusion of randomly selected ART clinics. The use of a more rigorous PN assessment tool that is considered as a gold standard for the assessment of HIV – associated PN ensured more reliable data to determine the prevalence PN in Rwanda. The data in this study can be used in developing strategies for the comprehensive management of the condition, particularly among urban and in the older, PLHIV on ART in Rwanda.

However, the study’s major limitation is that participants were only outpatients and lacked a balance of patients at different stages of HIV which might have influenced the level of CD4 cell count mostly identified the sample. Additionally, few participants in the sample were on d4T containing regimen by the time of the study which may have underestimated the association of the d4T with PN, tested. Occupational activities were used to identify the lifestyle of the participants but a more rigorous assessment procedure to identify the lifestyle activities that may be influencing the occurrence of PN, would strengthen the study. The study is biased with the cross-sectional study design and with some retrospective data from the medical file records, whereby some data such PN prior ART initiation were mostly missing and the variable could not be included in final analysis. A cohort study design to assess the factors influencing the PN among the PLHIV would be essential to give more evidence. The study also is limited in the assessment of inter-observer variation between the three assessors.

## Conclusions

The study identified a high prevalence of PN in Rwanda and this can form the basis for designing strategies for managing the problem and to prevent or minimise the effects of PN that compromise the quality of life of PLHIV. Older PLHIV and living in urban settings, on ART should have their PN status monitored more so as to maintain and improve their quality of life appropriately. However, further study into lifestyle behaviour activities and other factors that may influence the occurrence of PN assessed longitudinal design is needed among PLHIV in urban settings.
